# Commentary: Diagnostic Accuracy of Blood-Based Biomarker Panels: A Systematic Review

**DOI:** 10.3389/fnagi.2022.895398

**Published:** 2022-04-29

**Authors:** Satyakam Bhagavati

**Affiliations:** Department of Neurology, State University of New York Downstate Medical Center, New York, NY, United States

**Keywords:** Alzheimer's dementia, tau, amyloid, biomarker, blood

## Introduction

Alzheimer's disease (AD), the most common cause of dementia, is defined on the basis of its underlying molecular pathology, the accumulation of extracellular amyloid plaques (amyloid β) and intracellular neurofibrillary tangles containing hyperphosphorylated tau and the ensuing neurodegeneration. These deposits can be detected most definitively by amyloid-PET and tau-PET brain scans and also by cerebrospinal fluid analysis. However the widespread use of these tests is difficult because of cost, limitations in radiopharmaceutical availability and the need to do lumbar punctures. Recent reviews (Hardy-Sosa et al., 2022; Teunissen et al., 2022) have reported that concentrations in blood of amyloid and phosphorylated tau proteins correlates with their corresponding CSF concentrations and also with brain amyloid and tau pathology as assessed by PET scans. Furthermore, it has been reported that these blood biomarkers can differentiate AD from other neurodegenerative conditions and normal individuals (Hansson, 2021). Based on this, it has been suggested that that these blood biomarkers may soon become powerful ways for early and precise diagnosis of Alzheimer's disease, for monitoring of disease progression and treatment effects (Teunissen et al., 2022).

However several factors suggest that caution should be exercised before more widespread use.

### Amyloid and Tau Deposits in Normal Elderly

Numerous neuropathological studies have shown that the hallmark pathological changes of Alzheimer's disease, amyloid plaques and neurofibrillary tangles containing hyperphosphorylated tau are not limited to individuals with dementia but are also present in the brains of cognitively normal older people. For example about 40% of cognitively normal people, autopsied at a mean age of 82–85 years met neuropathological criterion for Alzheimer's disease, with extensive diffuse and neuritic amyloid plaques and neurofibrillary tangles (Bennet et al., 2006). Similarly, amyloid-PET studies show that ~30% of all normal controls have brain amyloid deposits (40% positive at age 80) (Jansen et al., 2015). Also cross-sectional autopsy studies have shown ~75–80% of individuals at age 70–80 years have evidence of tau pathology (Braak and Del Tredici, 2015) and on tau-PET ~ 70 % of cognitively normal or minimally affected elderly (mean age 76 years) have tau deposits (Weigand et al., 2020). In addition, although some studies have reported an association of blood amyloidβ and phosphorylated tau (p181, p217, and p231) levels with the rate of cognitive decline (Verberk et al., 2020), it has been shown that the cumulative incidence of dementia in amyloid and tau positive cognitively unimpaired individuals in their seventies is <20% at 5 years and <50% at 14 years, suggesting these deposits are not strong predictors of cognitive decline (Vos et al., 2013).

### Co-morbid Pathology

An autopsy study of dementia patients showed that isolated Aβ plaques and tau deposits, without other pathology, was only seen in 20–30% of cases. The vast majority (70–80%) of dementia patients have significant comorbid brain pathology such as aberrant Lewy body α-synuclein aggregates, insoluble aggregates of TAR DNA-binding protein 43 (TDP 43) or cerebrovascular disease (Schneider et al., 2007; Karanth et al., 2020). Furthermore in patients with AD, APOE4 carriers are 2.5 times more likely to have quadruple brain pathologies (plaques, tangles, Lewy bodies, and TDP-43 aggregates) than noncarriers (Karanth et al., 2020).

### Minimal Effect of Reducing Aβ Brain Load

A number of Aβ-depleting therapies have been shown to effectively reduce Aβ load in brain but not to reduce cognitive decline. For example treatment with β-site-APP cleaving enzyme 1 (BACE1) inhibitors (Imbimbo and Watling, 2019) or infusions of aducanumab, a monoclonal antibody that selectively targets aggregated Aβ drastically reduces amyloid load (Sevigny et al., 2016) but has minimal effect on clinical decline (Haeberlein et al., 2020). Also the monoclonal antibodies gantenurumab and solanezumab reduced amyloid load but had no effect on cognitive decline (Alzheimer's Association, 2020).

## Discussion

Collectively this evidence suggests that although changes in levels of blood biomarkers may accurately reflect brain amyloid or tau burden (Table 1), this may be seen in many cognitively normal elderly individuals and unreliable in their predictive capacity for cognitive decline. Moreover there is considerable overlap in the levels of these blood biomarkers between AD and normal groups, which would make it difficult to use them as stand-alone tests for early diagnosis (Janelidze et al., 2020). Although it may be argued that those with alterations in blood levels of βamyloid and p-tau, even if they are cognitively normal at the time of evaluation, will eventually develop dementia, currently there is no definitive evidence to support that assertion.

**Table 1:** Current and proposed tests for accurate and early detection of Alzheimer's disease.

**Imaging biomarkers**	**CSF biomarkers**	**Blood biomarkers**
MRI Brain: Medial temporal atrophy 18FDG-PET scan: Posterior cingulate and temporal lobe hypometabolism Brain Amyloid-PET scan: Detects insoluble Aβ fibrils in extracellular brain amyloid plaques (initially in medial parietal and frontal cortex) Brain tau-PET scan: Detects insoluble hyperphosphorylated tau in neurofibrillary tangles (initially in medial temporal and parietal cortex).	Amyloid β1-42 levels and Amyloid β1-42/Amyloid β1-40 ratio: Decreased ~50% in those with cerebral Aβ pathology. Total tau: Increased pTau 181: Increased pTau 217: Increased	Amyloid β 1-42 level and Amyloid β1-42/1-40 ratio: Decreased ~20% in those with cerebral Aβ pathology. pTau 181: Increased pTau 217: Increased pTau 231: Increased

*CSF and blood concentrations of plasma tau phosphorylated at different sites (pTau181, pTau217, or pTau 231) are increased in Alzheimer's disease patients and CSF and blood concentrations of Amyloid β 1-42 and Aβ1-42/Aβ 1-40 ratio are decreased. Amyloid-PET brain scans are done with the PET-ligands 18F flutemetamol, 18F flobetapir or 18F florbetaben to detect insoluble Aβ fibrils in amyloid plaques. Tau-PET scans using ligands such as 18F flortaucipir bind to insoluble tau fibrils. Positive results on the blood tests have been validated by the corresponding results of PET scans which are considered definitive*.

In addition, because of the co-existence of multiple other dementia-causing brain pathologies in ~70–80% of patients, therapeutic decisions based on selective focus on the load of amyloidβ or tau pathologies, as reflected by levels of blood biomarkers, may be misdirected. If, using conservative estimates, even one-third of a cross-section of the population over 65–70 years of age have amyloid and tau deposits in their brain, using amyloid β and p-tau blood biomarkers for early detection of AD and treatment may place a colossal burden on health care services without benefit in most.

The use of proposed protein biomarker panels (such as Aβ42/Aβ40 ratio in blood, age, gender and APOE4 status) or other immune response and neurodegeneration biomarkers (such as antitrypsin, complement C3, different cytokines, neurofilament light chain, or glial fibrillary-acidic protein; Hardy-Sosa et al., 2022) may eventually prove of value but need further validation. Emerging insights into the role of processes upstream of both Aβ and tau, such as apoliprotein E, the endocytic system, cholesterol metabolism, and microglial activation should eventually complement blood biomarker data in better defining at-risk individuals.

## Author Contributions

The author confirms being the sole contributor of this work and has approved it for publication.

## Conflict of Interest

The author declares that the research was conducted in the absence of any commercial or financial relationships that could be construed as a potential conflict of interest.

## Publisher's Note

All claims expressed in this article are solely those of the authors and do not necessarily represent those of their affiliated organizations, or those of the publisher, the editors and the reviewers. Any product that may be evaluated in this article, or claim that may be made by its manufacturer, is not guaranteed or endorsed by the publisher.
